# Supplementary figures and images for: CENP-C functions in centromere assembly, the maintenance of CENP-A asymmetry and epigenetic age in Drosophila germline stem cells
Source: PLoS Genet. 2021 May 20;17(5):e1009247. doi: 10.1371/journal.pgen.1009247 (PMC8136707; doi:10.1371/journal.pgen.1009247)

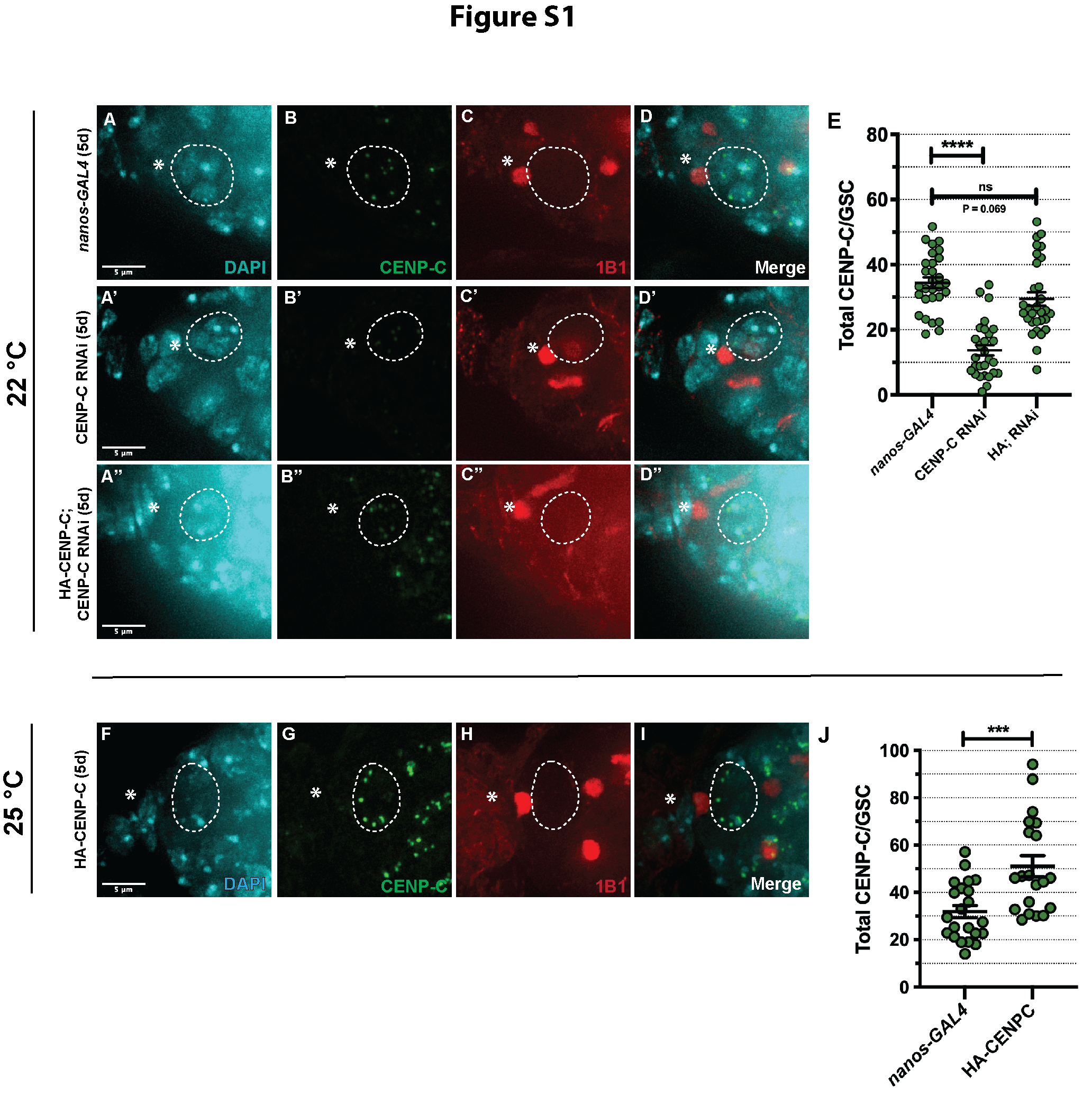

Supplement: S1 Fig — Immunofluorescent image of 5-day old (5d) G2/prophase GSCs (circled) in (A-D) nanos-GAL4 control, (A’-D’) CENP-C RNAi and (A”-D”) HA-CENP-C; CENP-C RNAi stained with DAPI (cyan), CENP-C (green) and 1B1 (red). Scale bar = 5 μm. CENP-C RNAi and rescue experiments were performed at 22°C. (E) Quantitation of total CENP-C fluorescent intensity (integrated density) per GSC in nanos-GAL4, CENP-C RNAi and HA-CENP-C; CENP-C RNAi (rescue). ****p<0.0001, ns = non-significant. Error bars = SEM. (F-I) Immunofluorescence image of 5-day old (5d) G2/prophase GSCs (circled) over-expressing HA-CENP-C stained with DAPI (cyan), CENP-C (green) and 1B1 (red). * denotes cap cells/GSC niche. GSCs are circled. Scale bar = 5 μm. HA-CENP-A over-expression experiments were performed at 25°C. (J) Quantitation of total CENP-C fluorescent intensity (integrated density) per GSC in HA-CENP-C. ***p<0.001. Error bars = SEM. (TIF) [file pgen.1009247.s001.tif]

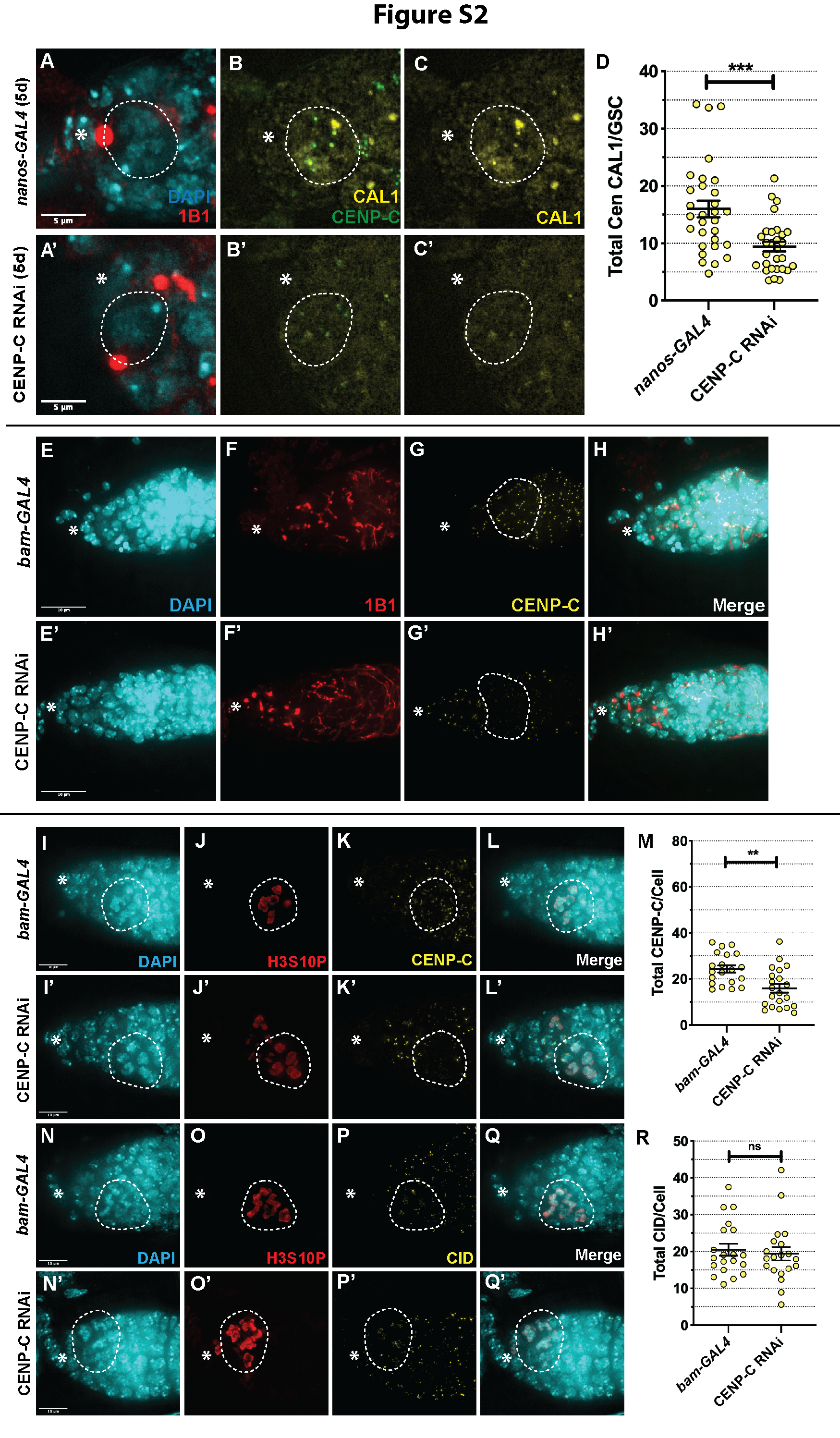

Supplement: S2 Fig — Immunofluorescent image of G2/prophase GSCs (circled) in (A-C) nanos-GAL4 and (A’-C’) CENP-C RNAi stained for DAPI (cyan), 1B1 (red), CAL1 (yellow) and CENP-C (green). Centromeric CAL1 was identified as being colocalised with CENP-C. GSCs are circled. *denotes cap cells. Scale bar = 5 μm. (D) Quantitation of total centromeric CAL1 fluorescent intensity (integrated density) in nanos-GAL4 and CENP-C RNAi. ***p<0.001. Error bars = SEM. (E-H) bam-GAL4 and (E’-H’) bam-GAL4 driven CENP-C RNAi stained with DAPI (cyan), CENP-C (yellow) and 1B1 (red). Circle marks region where knockdown begins. (I-L) bam-GAL4 and (I’-L’) bam-GAL4 driven CENP-C RNAi stained with DAPI (cyan), H3S10P (red) to mark 8-cell cysts in mitosis (circled) and CENP-C (yellow). (M) Quantitation of CENP-C in each cell of 8-cell cysts of bam-GAL4 and CENP-C RNAi. **p<0.01. Error bars = SEM. (N-Q) bam-GAL4 and (N’-Q’) bam-GAL4 driven CENP-C RNAi stained with DAPI (cyan), H3S10P (red) to mark 8-cell cysts in mitosis (circled) and CID (yellow). (R) Quantitation of CID in each cell of 8-cell cysts of bam-GAL4 and CENP-C RNAi. ns = non-significant. Error bars = SEM. * denotes cap cells. Scale bar = 10 μm. (TIF) [file pgen.1009247.s002.tif]

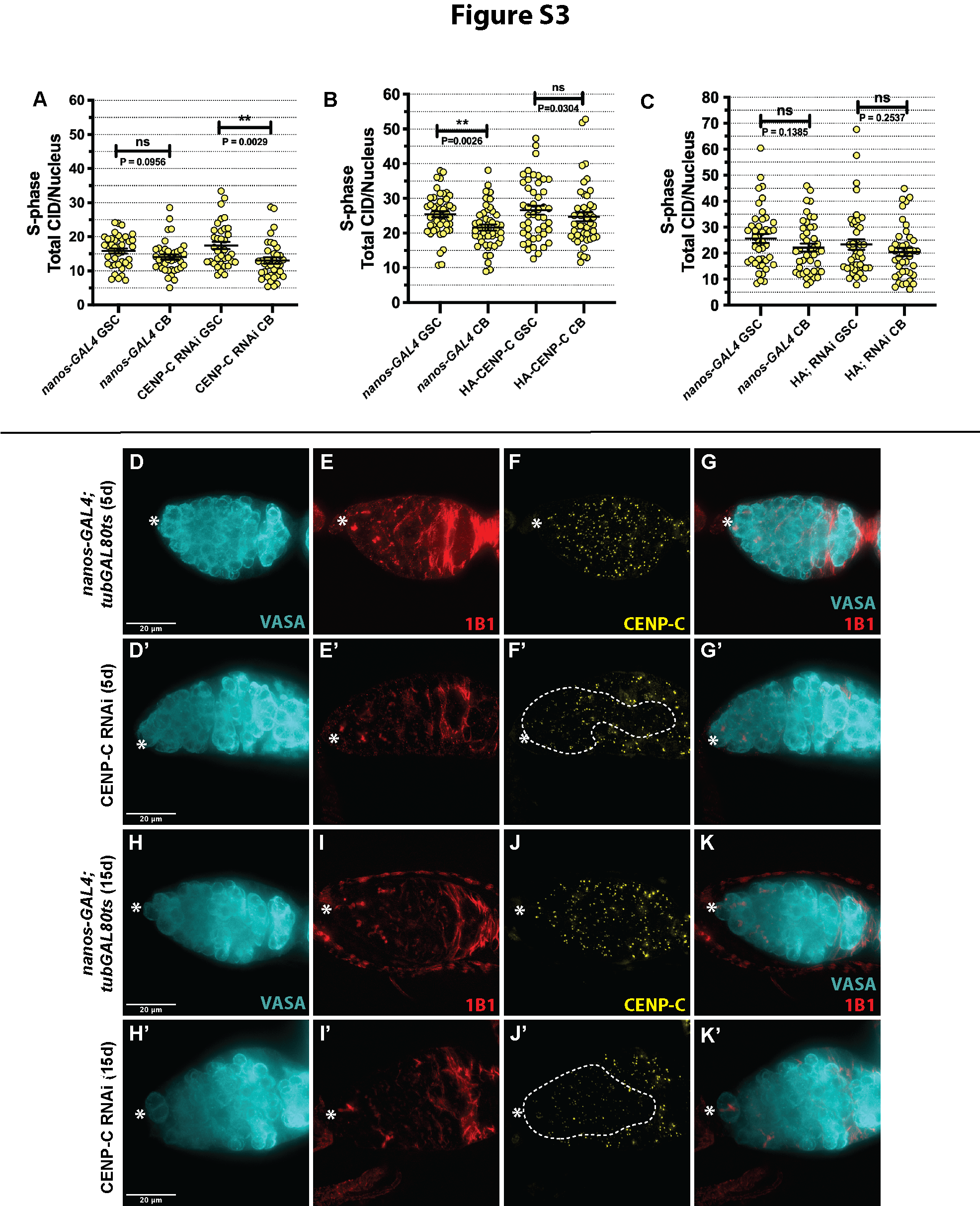

Supplement: S3 Fig — Quantitation of total CID fluorescent intensity (integrated density) in S-phase GSCs and CBs in nanos-GAL4 and (A) CENP-C RNAi or (B) HA-CENP-C or (C) HA-CENP-C; CENP-C RNAi (rescue). Each point represents the total CID integrated density per GSC/CB nucleus. **p<0.01. ns = non-significant. Error bars = SEM. (D-G, H-K) nanos-GAL4; tub-GAL80ts and (D’-G’, H’-K’) nanos-GAL4; tub-GAL80ts driven CENP-C RNAi stained with VASA (cyan), 1B1 (red) and CENP-C (yellow). Progeny were analysed at 5 (5d, D-G’) and 15 days (15d, H-K’) post eclosion. White circle outlines CENP-C depleted regions. * denotes cap cells. Scale bar = 20 μm. (TIF) [file pgen.1009247.s003.tif]

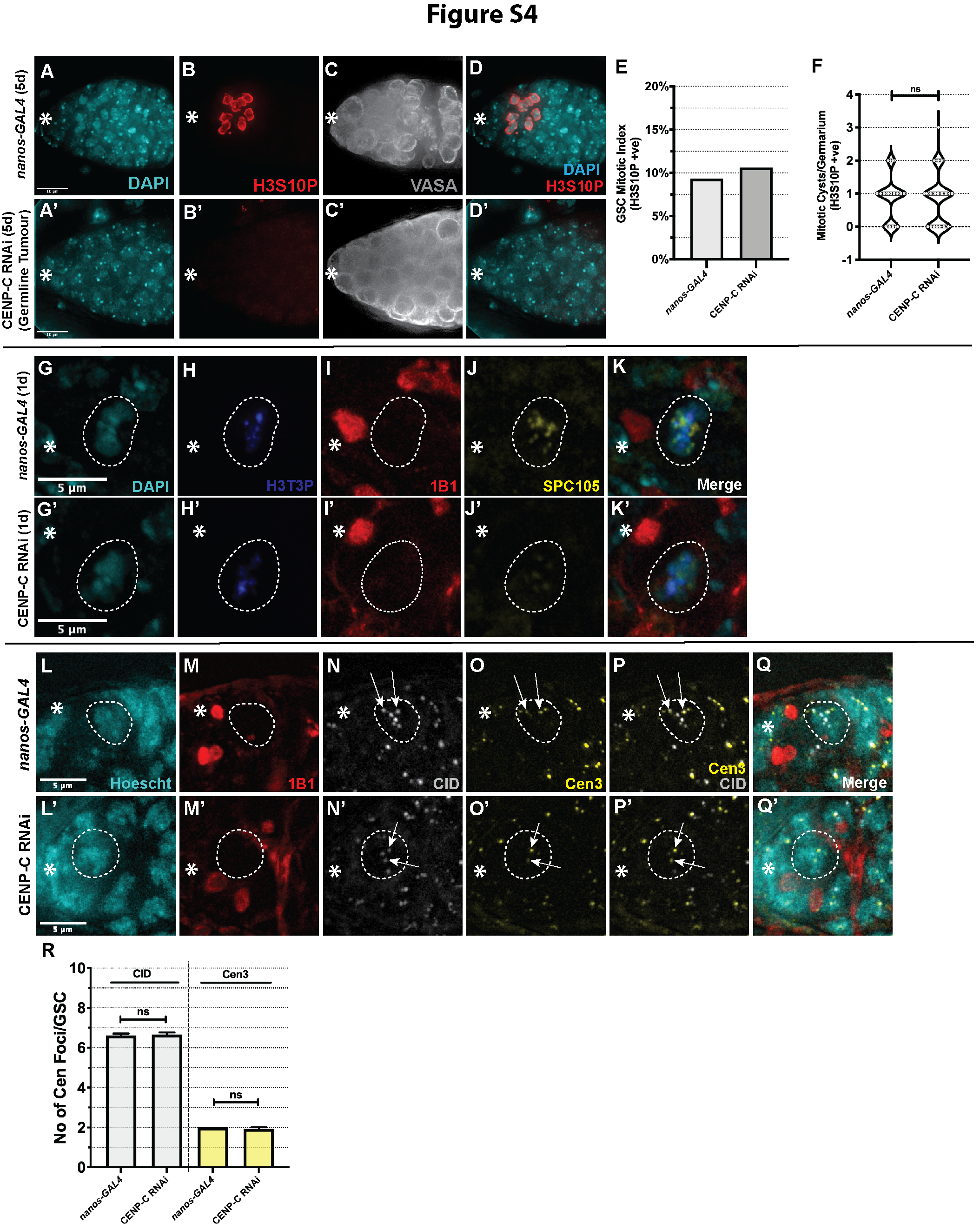

Supplement: S4 Fig — (A-D) 5 day old nanos-GAL4 and (A’-D’) CENP-C RNAi (germline tumour phenotype) stained with DAPI (cyan), VASA (grey) and H3S10P (red). * denotes cap cells. Scale bar = 10 μm. (E) Mitotic index (%) of H3S10P positive GSCs in nanos-GAL4 and CENP-C RNAi (n = 150 germaria). (F) Violin plot displaying the number of H3S10P positive cysts per germaria (n = 150 germaria). One positive hit was quantified as H3S10P positive GSC-CB pairs, 2-cell cysts (2cc), 4-cell cysts (4cc) or 8-cell cysts (8cc). ns = non-significant. (G-K) 1 day old nanos-GAL4 and (G’-K’) CENP-C RNAi stained with DAPI (cyan), histone H3 phosphorylated on threonine 3 (H3T3P) to mark prometaphase GCSs (blue), Spc105 (yellow) and 1B1 (red). 1 day old flies were analysed for this experiment in order to isolate prometaphase in actively dividing GSCs (circled). * denotes cap cells. Scale bar = 5 μm. (L-Q) nanos-GAL4 and (L’-Q’) CENP-C RNAi stained with Hoescht (cyan), 1B1 (red), CID (grey) and Cen3Giglio oligopaint FISH (yellow). White arrows indicate Cen3 that overlap with CID foci. GSCs are circled. (R) Quantitation of CID foci per GSC (grey bars) or Cen3 foci (overlapping with CID) per GSC (yellow bars) in nanos-GAL4 and CENP-C RNAi (n = 30 GSCs). ns = non-significant. Error bars = SEM. (TIF) [file pgen.1009247.s004.tif]

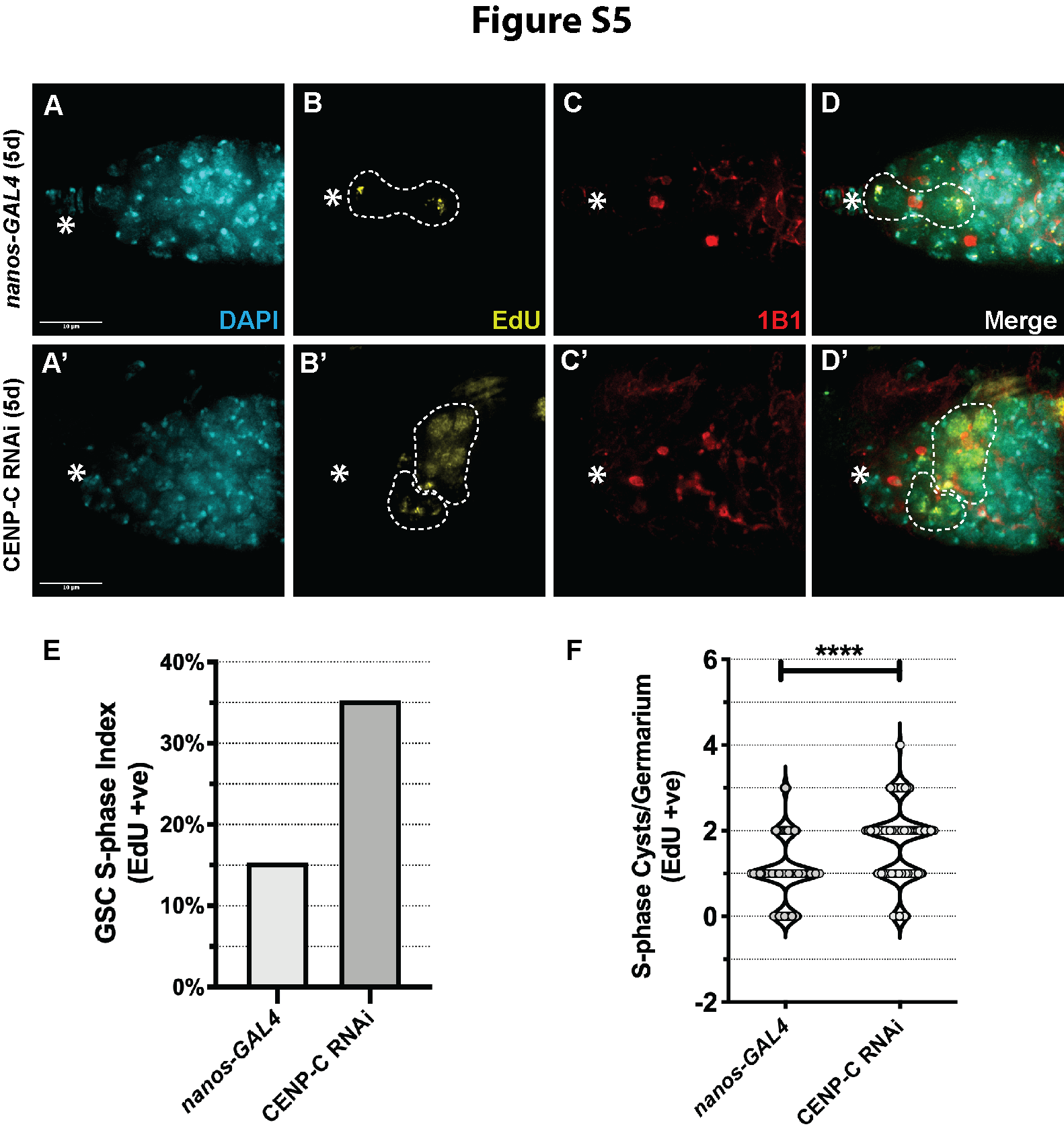

Supplement: S5 Fig — (A-D) 5 day old nanos-GAL4 and (A’-D’) CENP-C RNAi stained with DAPI (cyan), EdU (yellow) and 1B1 (red). * denotes cap cells. White dashed lines outline EdU positive GSC-CB (top) or cysts (bottom). Scale bar = 10 μm. (E) S phase index (%) of EdU positive GSCs in nanos-GAL4 and CENP-C RNAi (n = 150 germaria). (F) Violin plot showing the quantitation of EdU positive cysts per germarium (n = 150 germaria). One positive hit was quantified as a single EdU positive GSC/CB, 2-cell cysts (2cc), 4-cell cysts (4cc) or 8-cell cysts (8cc). ****p<0.0001. (TIF) [file pgen.1009247.s005.tif]

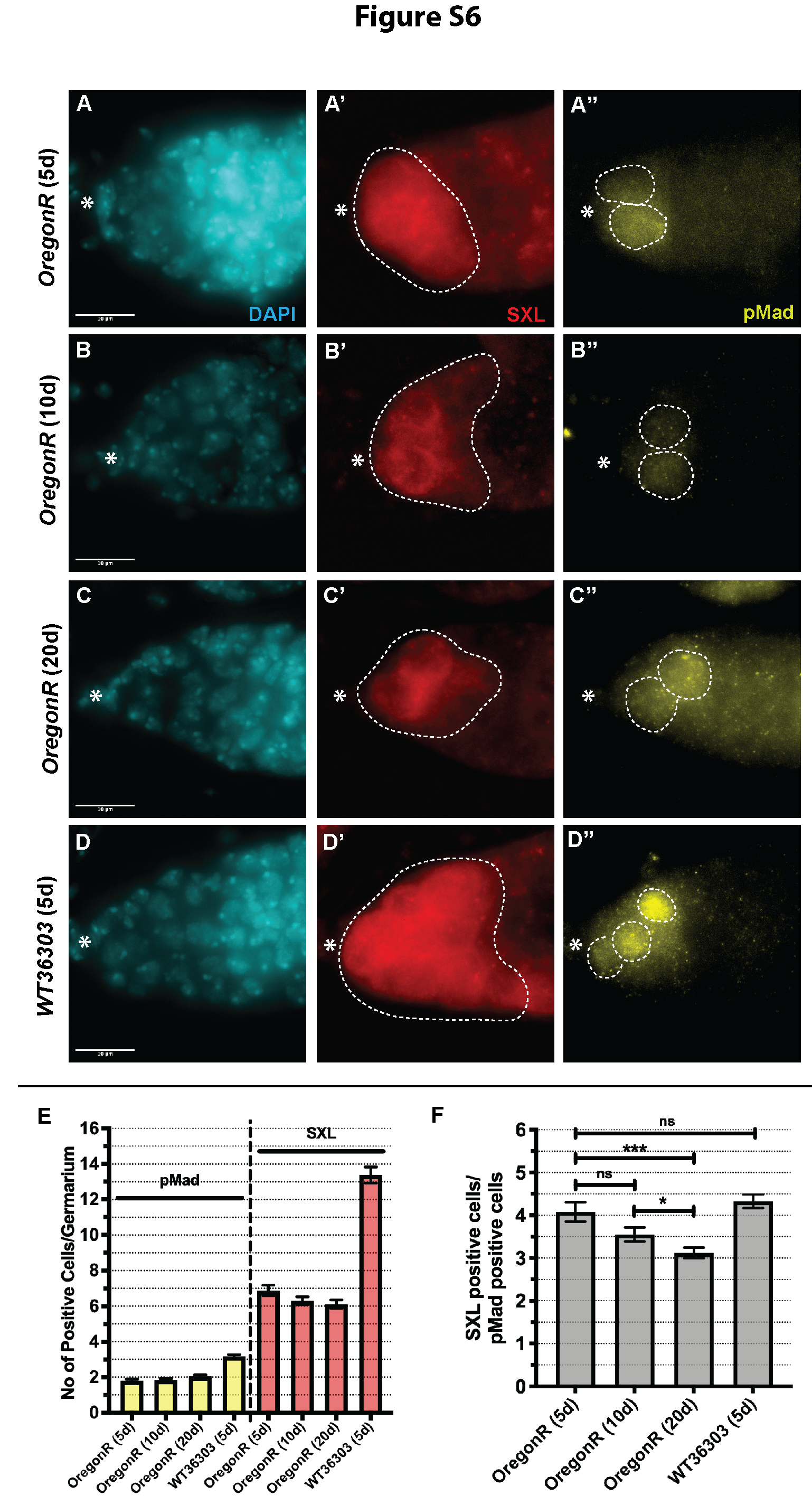

Supplement: S6 Fig — (A-C) Wild type (OregonR) (5-, 10- and 20-days old) and (D) wild type (#36303, RNAi isogenic control) germaria stained with DAPI (cyan), SXL (red) and pMad (yellow). *denotes cap cells. Scale bar = 10 μm. White dashed circles highlight SXL or pMad positive cells. (E) Quantitation of the number of pMad positive (left, yellow) and SXL positive (right, red) per germarium (n = 40–45). (F) Ratio of the number of SXL:pMad positive cells per germarium. ***p<0.001, *p<0.05, ns = non-significant. Error bars = SEM. (TIF) [file pgen.1009247.s006.tif]
