# Supplementary material for: CENP-C functions in centromere assembly, the maintenance of CENP-A asymmetry and epigenetic age in Drosophila germline stem cells
Source: PLoS Genet. 2021 May 20;17(5):e1009247. doi: 10.1371/journal.pgen.1009247 (PMC8136707; doi:10.1371/journal.pgen.1009247)
Supplement: Attachment — Submitted filename: Response to Reviewers_Carty et al.pdf [file pgen.1009247.s008.pdf]

Response to reviewers:

Reviewer #1: The manuscript entitled 'CENP-C regulates centromere assembly, asymmetry and epigenetic age in Drosophila germline stem cells' by Ben Carty et al. deals with the question how Drosophila CENP-A/CID is loaded and maintained in female germ line stem cells. Like in other cells types of Drosophila melanogaster, the authors show that CENP-C is essential for CENP-A localization to the germ line stem cells and its assembly at G2 phase of the cell cycle. In addition, CENP-C is also required for the asymmetric distribution of CENP-A. The authors find that in aging germ line stem cells with reduced CENP-C, CENP-A decline is more rapid. Last but not least CENP-C also seems to be involved in regulating the asymmetric division of germ line stem cells with a higher percentage of symmetric division and, therefore, self-renewal of germ line stem cells.

The manuscript is well written and addresses interesting aspects of centromere biology and reproductive biology also in the context of aging. I would like to raise several points that the authors may want to consider for improving their findings:

It isn't always clear how the authors distinguished cells in S phase and G2 phase. I understand that it is based on the EdU staining and the spectrosome but the EdU is hard to see because of its overlap with the DAPI staining. A separate panel with EdU staining only should be included. In addition, the spectrosome does not always look extended but sometimes more like a round spectrosome (Fig. 1C, 2A). The quantifications look good and I assume that looking at the actual samples makes it much easier to distinguish it but the authors should choose pictures that reflect what they describe in the text. Including other markers (for instance Vasa would also improve the images and could serve as a signal to normalize CENP-C/CENP-A/CAL1 on, see also further down).

For Figures 1, 2 and 3, we now show individual channels for EdU and 1B1. In Figure 1, we use arrows to clearly indicate the typical spectrosome shape for S phase (bridge) or G2 phase (round). We have also added a more detailed description to the text of how we identified GSCs in S phase and in G2 phase, referring to our previous publication that developed and used this method (Dattoli et al. 2020).

The quantification of Figure S2D isn't clear. The authors write that they quantified the centromeric CAL1 levels in control and CENP-C depleted cells. The centromeres are, however, defined here by CENP-C which is absent in the depletion. How can the authors measure Cal1 at centromere (and distinguish it from the nucleolar pool).

We measured an approximate 60% reduction in CENP-C in RNAi experiments. Therefore, CENP-C foci are still detectable after RNAi (although at a reduced level). We used this remaining CENP-C signal (green foci visible in Figure S2B') to identify the centromeric CAL1 signal for quantitation. We have clarified this in the text as follows: *Using residual CENP-C signals to mark centromeres, we quantified total centromeric CAL1 in GSCs at G2/prophase…*

Quantifying the signal of CENP-A in later developmental stages in control and CENP-C-depleted germarium would be good: The authors say that there is no effect on CENP-A, the provided images looks like there is even more CENP-A (S2J'). In addition, the authors should consider that at later developmental stages the significant amount of remaining CENPC (?, bam-driven depletion has not been quantified, should be done) is CENP-C-depleted cells may be sufficient for those cells to function (or that the RNAi isn't as effective as in earlier stages).

We have added a quantitation of CID and CENP-C level in the *bam-Gal4*-driven CENP-C RNAi now shown in Figure S2I-R. For this we used the H3S10P mitotic marker to identify

synchronously dividing 8-cell cysts in which the bam driver is active and therefore CENP-C should be knocked down. We then quantified CENP-C or CID intensity at this stage. We confirm a 40% reduction in CENP-C (Fig S2M), with no significant reduction in CID (S2R). We describe these results as follows: *Using phosphorylation at serine 10 of histone H3 (H3S10P) staining to identify synchronously dividing 8-cell cysts in mitosis, we quantified either total CENP-C or CID level per nucleus. In the CENP-C RNAi, we confirmed a 40% reduction in CENP-C (Fig S2M), however no significant change in CID intensity was measured between the knockdown and bam-GAL4 control (Fig S2R). This finding is comparable to our previous observations for CID and CAL1 [20], and indicates that a 40% reduction in CENP-C does not alter CID assembly in later divisions occurring in the germarium.*

In Figure 3 again, the EdU staining is hard to see.

As described above, we now show individual EdU and 1B1 channels to clarify the staining pattern of these cell cycle markers. EdU is pan nuclear in mid to late S phase; the spectrosome forms a bridge in S phase.

Figure 4. The presentation could be improved. The arrow with time suggests that the different phenotypes are derived from each other, which I don't think they do nor that the authors wanted to claim this. I would remove the arrow. Importantly, the fact that the germ line tumors are not rescued at all by CENP-C rescue experiments indicates an unresolved proliferation defects cause by too much CENP-C or CENP-C expressed at the wrong time of the cell cycle that the authors should address further, at least in the discussion if not experimentally. The claims in the result section need to be toned down somewhat and this phenomenon discussed.

We have removed this arrow as we do not know if these phenotypes are derived from each other. The reviewer raises a good point that the germline tumour phenotype is not rescued by CENP-C over-expression. We now address this point in the results as follows: *Notably, HA-CENP-C over-expression did not rescue the germline tumour phenotype, possibly indicating that excess CENP-C or the incorrect timing of CENP-C expression or turnover can lead to proliferation defects.*

The increased number of EdU positive cells in CENP-C RNAi germ cells where interpreted as 'going through S-Phase slower' when CENP-C is depleted. However, could it also be an S-phase bloc that the authors observe?

We have modified this statement to read: *This suggests that GSCs and cysts in the CENP-C RNAi are either blocked or progress slower through DNA replication in S-phase.*

How can the graph presented in S4Q (error bars!) be highly significant?

The error bars on the previous graph represented the max and min and the median value was shown. We have replaced this graph with a violin plot showing the all data points, as we believe this is a better way to represent the data (now shown in Figure S5F). Between 0 and 3 EdU positive cysts in replication were counted in the control, whereas between 0 and 4 were counted in the CENP-C RNAi. We calculated significance based on the mean value. We have also added a count of the S-phase index for GSCs (based on EdU positivity, n=150 germaria) and report that a higher percentage for the CENP-C RNAi (35%) compared to the control (15%), now shown in Figure S5E.

In Fig 5, the authors could have made a more convincing claim if they would have also used readily available stem cell markers such as Vasa. Also, co-staining with a marker that does not change would have controlled for experimental variability and would have given the authors a signal to normalize the fluorescence intensity of CENP-C.

For quantitations shown in Figure 5, we also now use 1B1 staining as an internal control to normalise CID and CENP-C intensities at each timepoint and have added this detail to the methods section. After normalisation, the overall downward trend in intensity for CID and CENP-C over time is unchanged. To improve the presentation of Figure 5, we now show each channel individually, as well as the merged image. This allows us to more clearly indicate the selection of GSCs for analysis (with a round spectrosome, attached to the cap cell).

Reviewer #2: This work by E. Dunleavy and colleagues investigated whether CENP-C has a role in maintaining the "mitotic drive" to bias sister chromatid segregation, a novel phenomenon recently reported by several labs including the authors'. The authors are addressing an important question to further understand the underlying mechanisms to enhance our understanding of this phenomenon using Drosophila female germline stem cells as the model system. Importantly, they have shown that CENP-C, an inner kinetochore component, is required to maintain a normal asymmetric distribution of CID and GSC normal function. The approaches take advantage of the powerful molecular genetics and cell biology tools, which are in general well executed. However, some experiments will need additional control and some results will need additional analyses.

Major comments:
1. Throughout the manuscript for the RNAi experiments, the control is using the nanos-GAL4 driver only, it is better to use a control of nanos-GAL4 paired with some non-specific RNAi to activate the RNAi pathway, such as UAS-lacZ RNAi or GFP RNAi, etc. However, for some sets of experiments, the rescuing experiment was performed which showed more gene specificity, it may not be necessary to repeat those experiments when the rescue experiment was performed.

In our previous publication (Dattoli et al. 2020, JCB), we included an experiment in which mCherry-RNAi was driven by the same *nanos-GAL4 driver* which we have used for all experiments in this study and did not observe any phenotype in germaria.

2. Again, for the RNAi experiments, the authors argued that the secondary effect should be minimal. But normally to make the knockdown acute, it is better to combine the nanos-Gal4 driver with the tub-Gal80_ts in order to knockdown at a particular stage, such as adulthood, in order to prevent prolonged knocking down and potential secondary defects.

We thank the reviewer for the suggestion to try the *nanos-Gal4* driver in combination with the *tub-Gal80_ts* in order to improve temporal control. We now include a CENP-C-RNAi experiment using this driver (gift from Yukiko Yamashita) in Figure S3D-K'. We analysed the progeny at 5 and 15 days after the temperature shift and RNAi induction and could confirm CENP-C knockdown at both timepoints. At both 5 and 15 days, we noted the germline tumour phenotype. Importantly, we now show that the SXL/pMad ratio is also reduced after 15 days (shown in Figure 6G-H'', I and J). Taken together, we conclude that the spectrum of phenotypes we observed in Figure 4 need more time to manifest i.e. knockdown in GSC precursors upon *nanos* expression in early embryogenesis is important. Notably, the *nanos-Gal4* driver (#25751) has been used in previous RNAi screens for GSC maintenance in females (Yan et al. 2014, Developmental Cell), so we believe it is a valid approach to drive UAS-RNAi transgenes in GSCs and to reveal associated phenotypes. We now refer to this study in the results section also.

3. In their previous paper, they showed difference of CID between sister centromeres which is very nice and avoid any complication of cell cycle stage difference. In this manuscript, all data are exclusively in post-mitotic GSC-CB pairs. Examination of changes at individual sister centromeres would be very informative.

In our previous publication, we used superresolution (SIM) microscopy for this analysis. These experiment we performed at the University of Edinburgh Superresolution Imaging Consortium. Unfortunately, due to the pandemic, it is not possible for us to travel to Edinburgh to complete this task. Instead, we have provided example images showing asymmetric CENP-C distribution at very early anaphase and at telophase, acquired using our DV Elite widefield and deconvolution microscopy (Figure 3A-D'). Quantitation of CENP-C intensity revealed an approximate 1.4 fold asymmetric distribution between GSCs and CBs.

4. Centromere proteins are known to be critical to maintain normal mitotic progression and cell cycle progression. Therefore, prolong depletion of centromeric proteins could lead to GSCs loss. And this loss could be caused by failure in GSC self-renewal, or de-differentiation process, a phenomenon reported in this system especially during aging. Therefore, it would be interesting to explore whether the change of GSC number upon CENP-C depletion could be due to dedifferentiation defects.

We thank the reviewer for this interesting suggestion. De-differentiation in female GSCs was first shown by Kai and Spradling in 2004 using an anti-Anillin antibody to detect closed or remnant ring canals. This antibody is not available commercially, however we did carry out a staining with anti-hu-li tai shao (Hts-RC) to detect ring canals. We found that CENP-C depleted germaria were comparable to controls. We have not included this experiment in our revised manuscript as we believe it requires further analysis that is beyond the scope of this study. Instead, we have added the following sentence to the discussion: *At this point we cannot exclude the possibility that these GSCs might result from dedifferentiation, which can occur in Drosophila germaria (Kai and Spradling, 2004).*

Minor comments:
1. The sentence "We found an increase in CENP-C level between cells in S-phase, compared to cells in G2/prophase." is confusing, should be revised to: We found an increase of CENP-C level in G2/prophase cells compared to cells in S-phase.

We have fixed this.

2. For this conclusion "This result indicates that CENP-C is specifically required for CID assembly that occurs between the end of S-phase up to prophase in GSCs.", it may be a bit overstretch given the temporal resolution the EdU label can inform.

We have modified this conclusion to read: *This result indicates that CENP-C is specifically required for CID assembly that occurs between S-phase and prophase in GSCs.*

3. Fig. 2J and 2K, the statistical analyses should also be performed at the comparable cell cycle stage between genotypes, in order to support conclusions such as "no significant change" or "partial rescue". Similarly, in Fig.5M, the statistical analyses should also be performed at the comparable age between genotypes.

We have added these statistical analyses to the graphs 2J, 2K (now called 2Q, 2W) and 5M.

4. Page 7, "we assayed whether cells were blocked in mitosis using phosphorylation at serine 10 of histone H3 (H3S10P) to marks cells at late G2-phase to metaphase." I guess the authors mean M phase instead of metaphase? H3S10P is detectable throughout M phase including anaphase and telophase.

Yes, we have corrected this to read 'late-G2 phase and mitosis'.

Also, this mark was labeled incorrectly in Figure S4, with a pattern more like at the periphery of the chromosomes?

We confirm that Figure S4B does show H3S10P staining. This is the typical pattern we observed for H3S10P in 8-cell cysts.

Finally, it is said in the text that "H3S10P combined with DAPI staining of DNA allowed us to monitor for chromosome segregation defects, which were also absent (data not shown)." It would be better to add data in the supplement. The mitotic index would inform any potential mitotic defects. For example, there is no mitotic GSCs in Figure S4, which could be due to cell cycle arrest. In addition, GSCs at anaphase and telophase would be the best stage to examine whether there is any segregation defect.

We have now added a count of the mitotic index for GSCs (Figure S4E) and a quantitation of the number of mitotic cysts per germarium (S4F) to Figure S4. We did not find any significant increase in the CENP-C RNAi. To further address potential chromosome segregation defects, we have added staining for the Spc105/KNL1 inner kinetochore marker. We find that Spc105 still localises at centromeres at prometaphase in the CENP-C RNAi, but at a reduced level. This data is now included in Figure S4G-K'. To detect aneuploidy, we have quantified CID foci per GSC, as well as Cen3 FISH signals per GSC (that overlap with CID) and did not find any significant difference between the control and CENP-C RNAi. This data is now included in Figure S4L-R.

5. For this statement in the text on page 8, "Given that symmetric GSC divisions (in which the CID ratio is presumably 1) occur at a low frequency [37,38], we hypothesised that CID and CENP-C levels would gradually be depleted in GSCs over time (Fig 5)." I do not quite understand the rationale here.

To improve clarity, we have modified this statement as follows: *Wild type GSCs retain 1.2-fold more CID in an asymmetric division. However, given that symmetric GSC divisions (in which the CID ratio is presumably 1.0) also occur [37,38], we hypothesised that CID and CENP-C levels would gradually change in GSCs over time (Fig 5).*

6. The pMAD staining signals in Figure 6A" and S5C"and S5D" have lots of background, which would make it hard to count the exact number of positive cells for this marker.

We agree that anti-pMad antibody staining can show a lot of background. The images shown in Figures 6 and S6 are projections of the number of z stacks that capture the entire GSC. For our quantitations, we always carefully scanned each z-stack series to identify GSCs that were pMad positive and also SXL positive. We have clarified this in the methods section: *Quantification of pMad and SEX-LETHAL positive cells was obtained by scanning the z-stack of each image to count cells with specific signals and to distinguish from any background signals.*

In Figure 6 legend: *Images are projections of z-stacks that capture total pMad/SXL signal per germarium.*

7. Page 10 in Discussion, "Ultimately, our findings for CENP-C function in GSCs are in full agreement with the mitotic drive model for stem cell regulation [9]." Wrong reference here.

We have fixed this reference.

Reviewer #3: Previous work has shown some unusual characteristics of centromere protein loading in germ line cells. Previous work from the Dunleavy lab has shown that centromere protein CENP-A/CID is deposited during G2. In addition, it is partitioned unevenly between

centromeres following replication. One possible implication of this is that sister kinetochores do not randomly attach to microtubules, leading to non-random segregation of chromatids and possibly effects on differentiation. This paper focuses on analysis of CENP-C in germ line divisions using two important tools, RNAi to Cenp-C and an RNAi-resistant Cenp-C transgene.

The results are solid with carefully constructed controls. For example, there are separate sets of nos-Gal4 controls in Fig 3. In addition, most of the images are nicely presented, and the centromere signals are easy to see. However, it can be a struggle to see the 20% differences represented in the graphs. For example, the image in 1D' is apparently brighter than 1C'. Not sure there is a solution to this.

All our quantitations represent the sum fluorescence of all centromeres per nucleus. We have replaced this image with a S-phase and G2-phase cells that more clearly display signals representative of our quantitations. For Figures 1, 2 and 3, we now show individual panels for 1B1 and EdU, which we hope clarifies the cell cycle stages (see response to point 1 of Reviewer 1 also).

The results are interesting, but many are not surprising based on previous studies on CID recently published in 2020. The results in the first part of this paper show CENP-C behaving much like CID and Cal1. The more interesting results are the germline proliferation defects in CENP-C RNAi. However, there are some concerns because the data hints that there is a defect in S-phase but it is not clear what that is, and the authors do not do a good job of ruling out chromosome segregation defects. These points and additional less significant issues are discussed below.

1) The first set of major findings is that CENP-C shares the unique properties of CID in the germline. It is required for increased CID in G2, and this can be rescued with an RNAi resistant transgene. Overexpression of Cenp-C, however, has little effect on asymmetric CID, suggesting the levels of CENP-C don't drive this process. Also like CID, CENP-C is asymmetric between the GSC and CB cells. A little surprising result is that CENP-C depletion increases the GSC-CB ratio, possibly indicating that when CENP-C is limiting, preference goes to loading in the GSC.In this section, the authors should be careful not to overstate the significance of their results. Given the known function of CENP-C to interact with CAL1 and CID in centromere, it is not surprising that, like the previous 2020 publication, CENP-C has a role in G2 loading and asymmetric assembly of CID. Perhaps the authors should discuss whether the role of CENP-C observed is via the known pathway of centromere assembly, or is it modified to result in G2 loading and/or asymmetric inheritance. More importantly, the authors should be careful not to overstate their results and suggest a direct function of CENP-C in regulating G2 loading and asymmetric inheritance. The heading on pg 6 suggests CENP-C activity promotes asymmetry.

The last line of pg 9 makes a similar conclusion. However, observing an effect of CENP-C depletion on G2 loading and asymmetric inheritance is only consistent with a role in centromere assembly. Instead, these sentences imply that CENP-C is responsible for these unique features. To show a direct role of CENP-C, the authors would need to have a separation of function result and show that a specific CENP-C depletion effects only the G2 loading or asymmetric inheritance, while other functions (like building centromeres and kinetochores) are not affected. In fact their results argue the opposite. Overexpression of CENP-C did not affect the asymmetric behavior of CID. Doesn't this argue against a role of CENP-C in regulating asymmetry? (as opposed to the results with overexpression of CID or CAL1 in the 2020 paper).

We thank the reviewer for these insightful comments. We agree that our data supports CENP-C's function in CID assembly in GSCs. Upon CENP-C depletion, CID asymmetry is disrupted i.e. becomes even more asymmetric, yet over-expression of CENP-C does not

appear to drive asymmetry. Therefore, CENP-C may not function directly in asymmetry. Rather CENP-C might function differentially to maintain CID in stem and daughter cells. We have modified the results and discussion sections as follows to accommodate this possibility.

- We have changed the title on p6 to: *Reduced CENP-C increases CID asymmetry between GSCs and CBs.*
- We have toned down our interpretation of these results on p7 as follows: *This indicates that in addition to CID assembly in G_{2}/prophase, CENP-C potentially functions in maintaining CID asymmetry in S-phase.*
- At the end of this section on p7: *Taken together, these results show that at 5 days old although CID asymmetry is perturbed after CENP-C RNAi, supply of excess CENP-C is not sufficient to drive CID asymmetry in stem and daughter cells at S-phase*.
- In the discussion: *In addition to its function in assembly, we find that CENP-C is required to maintain the correct level of CID asymmetry between stem and daughter cells. Specifically, depletion of CENP-C gives rise to GSCs retaining 1.44-fold more CID compared to 1.2 in the controls. Given that CENP-C over-expression was not sufficient to drive CID asymmetry, we suggest that CENP-C's function in asymmetry is likely due to its canonical role in CID assembly. CENP-C might function differentially to maintain CID in GSCs and CBs. It is also possible that CENP-C functions directly in establishing CID asymmetry.*
- We have also modified the manuscript title to reflect CENP-C's function in the maintenance of asymmetry: *CENP-C functions in centromere assembly, the maintenance of CENP-A asymmetry and epigenetic age in Drosophila germline stem cells.*

2) The more novel results concern the role of Cenp-C in germ cell differentiation RNAi. CENP-C RNAi has a variety of germarium phenotype, and these get more severe with age. This is interesting but can the authors relate this to the biology a little more and discuss why severity increases with age? Nos-Gal4 expression begins in the germline. Does the age effect reflect the time it takes for CENP-C to be depleted?

Our new experiment of CENP-C RNAi controlled by the *nanos-Gal4* driver in combination with the *tub-Gal80_ts* has allowed us to knockdown CENP-C in adult GSCs (shown in Figure S3D-K'). We analysed the progeny 5 and 15 days after the temperature shift and RNAi induction and could confirm CENP-C knockdown at both timepoints. At both 5 and 15 days, we noted mostly the germline tumour phenotype. Yet, when we measured the SXL/pMad ratio, we found that consistent with our other CENP-C RNAi experiments, this ratio was significantly reduced compared to the control (Fig 6G-H'', I, J). Taken together, we conclude that the spectrum of phenotypes we observed in Figure 4 need more time to manifest i.e. knockdown in GSC precursors upon *nanos* expression in early embryogenesis is important. CENP-C knockdown at the adult stage is sufficient to perturb the SXL/pMad ratio and germ cell tumours are apparent. Notably, the *nanos-Gal4* driver (#25751) has been used in previous RNAi screens for GSC maintenance in females (Yan et al. 2014, Developmental Cell), so we believe it is a valid approach to drive UAS-RNAi transgenes in GSCs and to reveal associated phenotypes. We now refer to this study in the results section also.

What is the effect on fertility? Are the 5d and/or 10d females fertile? Do they produce embryos?

We have not performed fertility tests at this time. However, we can confirm that CENP-C RNAi germaria continue to develop egg chambers containing mature eggs. We now refer to this in the text and show representative images for each phenotype (Figure 4F-I).

The germ line tumor phenotype is also interesting but not explained. This seems to be the results that most strongly implicates CENP-C in differentiation.

We also now provide additional details of the germline tumour phenotype as follows: *However, another third (32%) displayed an accumulation of germ cells, indicative of a proliferation defect consistent with germline tumours [36].*

Moreover, as suggested by Reviewer 1, we note that HA-CENP-C overexpression does not rescue the germline tumour phenotype. We discuss this in the results section: *Notably, HA-CENP-C over-expression did not rescue the germline tumour phenotype, possibly indicating that excess CENP-C or the incorrect timing of CENP-C expression can lead to proliferation defects.*

Does Cal1 or CID knockdowns have these phenotypes or is this specific to CENP-C?

In our previous publication, we performed CAL1 and CID knockdowns in GSCs, which resulted in empty germaria. We now refer to this in the text: *Different from previous CID and CAL1 knockdowns that resulted in empty germaria (Dattoli et al. 2020), CENP-C depleted germaria revealed a spectrum of germ cell proliferation phenotypes…*

At the bottom of page 7 the authors conclude that there are no chromosome segregation defects. However, the data is “not shown”. While this result is plausible, given the partial KO of CENP-C and the tumor phenotype, this data needs to be shown since it is not clear how with just H3S10P staining this can be concluded. Instead, the author find that the depletion of CENP-C has an effect on S-phase progression. It would be really interesting if the authors have discovered a new S-phase function for CENP-C in germ line differentiation. However, they have to rule out mitotic defects, which would require more careful analysis such as mitotic index, karyotyping, and staining to markers like kinetochore ands checkpoint proteins. In short, this seems like a missed opportunity to show that partial loss of CENP-C affected differentiation but not cell division.

We have addressed the possible occurrence of chromosome segregation defects in CENP-C RNAi as follows:

- We provide the observed mitotic index in GSCs and in germaria (Figure S4A-F). We do not find any significant difference between the control and CENP-C RNAi.
- We stain for the kinetochore marker Spc105/KNL1. We find that at prometaphase, it localises as expected, although at a reduced level (Figure S4G-K’).
- As indicators of aneuploidy, we count centromere number using CID IF and Cen3 oligoFISH (Figure S4L-Q’). We do not find any significant difference between the control and CENP-C RNAi (Figure S4R).

In summary, we did not observe segregation defects. We believe that a 60% reduction in CENP-C allows for its canonical kinetochore function. We state this in the text as follows: *These results show that the extent of CENP-C depletion (60% reduction) does not result in a mitotic arrest nor in obvious chromosome segregation defects, indicating that the canonical kinetochore function of CENP-C is maintained.*

With respect to S-phase progression, to strengthen this data, we have added a count of the S-phase index for GSCs (based on EdU positivity, n=150 germaria) and report that a higher percentage for the CENP-C RNAi (35%) compared to the control (15%), now shown in Figure S5E.

3) The methods state that RNAi experiments were done at 22deg. This is significant because UAS/GAL4 is typically stronger at higher temps. Why was 25 deg not used? The knockdown may have been stronger and more severe phenotypes observed. Why was 29 used for bam-Gal4 and 25 for HA-CENPC, and what temperature was HA-Cenp-C+RNAi?

All *nanos-Gal4* driven CENP-C RNAi experiments were carried out at 22ºC. When these experiments were performed 25ºC, this resulted in a strong CENP-C knockdown, with most germaria displaying the GSC loss phenotype, which was not useful for our analysis. We used 29ºC for *bam-Gal4* driven experiments to ensure a strong depletion of CENP-C, as we were initially surprised that there was no major effect on CID localisation. CENP-C overexpression experiments were carried out at 25ºC as this resulted in robust HA-CENP-C over-expression. Despite this we could only ever achieve a 40% reduction in CENP-C at 29ºC. However, HA-CENP-C rescue experiments were performed at 22ºC, in line with all RNAi experiments. For this reason Figures 3J and 3K are presented in separate graphs and statistical analysis cannot be performed (see point 11 below). We now clarify these temperatures in the methods.

Pg 5: 7 lines from bottom: What is the basis for concluding that CENP-C is "dispensable" for later divisions. In the RNAi genotype, CENP-C protein is still visible and only reduced 60% (pg 5). Is the 40% protein localization level because the RNAi is not efficient, or because the protein is very stable. In addition, and for this reason, the evidence does not support the conclusion that Cenp-C is dispensable for centromere assembly in later germarium divisions. The knockdown might be to mild, and bam expression may be too transient to effect CID levels.

We have added a quantitation of CID and CENP-C level in the *bam-Gal4*-driven CENP-C RNAi now shown in Figure S2I-R. For this we used the H3S10P mitotic marker to identify synchronously dividing 8-cell cysts in which the bam driver is active and therefore CENP-C should be knocked down. We then quantified CENP-C or CID intensity at this stage. We confirm a 40% reduction in CENP-C (Fig S2M), with no significant reduction in CID (S2R). We describe these results as follows: *Using phosphorylation at serine 10 of histone H3 (H3S10P) staining to identify synchronously dividing 8-cell cysts in mitosis, we quantified either total CENP-C or CID level per nucleus. In the CENP-C RNAi, we confirmed a 40% reduction in CENP-C (Fig S2M), however no significant change in CID intensity was measured between the knockdown and bam-GAL4 control (Fig S2R). This finding is comparable to our previous observations for CID and CAL1 [20], and indicates that a 40% reduction in CENP-C does not alter CID assembly in later divisions occurring in the germarium.*

4) Figure 6: Is the 10d SXL/pMAD ratio significantly lower than 5d? I am guessing not, and if so, should be stated as such and the conclusion in line 16 can't be made. In addition, is the shift towards stem cells in 10d females (last line of section) a result of an arrest in cell division (either due to S-phase or mitotic defects).

We now indicate on the graph that the SXL/pMAD ratio was not significantly different between 5 days and 10 days in the *nanos-GAL4* control. We state that the balance of stem and daughter cells in the niche changes with age specifically after 20 days (presented in Figure S6F).

5) Pg 4, middle – Female GSCs divide… to give a differentiating (CB) and another GSC.

We have corrected this.

6) Pg 4. It would help to have more description of how you know the EdU-negative cells are in G2 versus G1 (Figure 1). Also, 4 lines from bottom, it might sound better to write that CENP-C levels were higher in G2 than S. The current sentence could be mis-interpreted.

To clarify EdU and 1B1 cycle cycle staining patterns we now show individual channels. We also refer to our previous publication (Dattoli et al. JCB) in which markers for the cell cycle stages were extensively characterised.

We have revised this statement as follows: *We found an increase in CENP-C level in G_2/prophase cells compared to S-phase cells.*

Pg 5, 7 lines from bottom: "occurring in the germarium"

We have corrected this.

7) Pg 6, line – in addition to reference to Fig 3A, it would help to report the GSC-CB ratio.

We have added this information: *In S-phase, we again confirmed 1.2 fold asymmetry for CID in nanos-GAL4 (Fig 3E, 3I).*

8) On pg 10, the authors state: "distorting the CID asymmetry disrupts the balance of stem and daughter cells". Has this been shown, or is it a correlation? If so, what is the evidence? Similarly, later on pg 10 "It is tempting to speculate that CENP-C …might be utilized to maintain parental CANP-A in a asymmetrically …". What data supports the idea that CENP-C maintains asymmetry, as opposed to being required for the loading process which is asymmetric.

To include these possibilities, we have modified this section of the discussion as follows:

In any case, it appears that distorting CID asymmetry (to either 1 or 1.4) correlates with a disrupted balance of stem and daughter cells in the ovary.

It is tempting to speculate that in addition to canonical functions in centromere assembly, CENP-C and/or CAL1 might be utilised in S-phase to establish or maintain CENP-A asymmetry in stem cells.

9) The sentence on pg 10 line 20 sounds interesting , but is not backed up by any data and should be deleted if the data is not shown.

We now refer to Figure S5 in support of this statement.

10) Pg 11, line 11, "CID levels"

We have corrected this.

11) In Figure 3, is HA; RNAi (3G) significantly different than HA without RNAi (3F)?

We could not calculate significance in this case as these experiments were performed at two different temperatures, each with a matched control (now Figs 3J and 3K). Importantly, the value for the *nanos-Gal4* control (1.2) was comparable for each experiment, therefore we do not believe that temperature impacts on CID asymmetry in the control. We clarify in the methods section that HA-CENP-C over expression studies were carried out at 25°C, whereas as CENP-C RNAi/rescue experiments were carried out at 22°C.
