# Supplementary material for: CENP-C functions in centromere assembly, the maintenance of CENP-A asymmetry and epigenetic age in Drosophila germline stem cells
Source: PLoS Genet. 2021 May 20;17(5):e1009247. doi: 10.1371/journal.pgen.1009247 (PMC8136707; doi:10.1371/journal.pgen.1009247)
Supplement: Attachment — Submitted filename: Response to Reviewers_Revision2.docx [file pgen.1009247.s009.docx]

We thank all 3 reviewers for their positive assessment of our revised manuscript.

Response to Reviewer #1:

The revised manuscript now entitled ’ CENP-C functions in centromere assembly, the maintenance of CENP-A asymmetry and epigenetic age in Drosophila germline stem cells.’ By Carty et al., has improved the manuscript and the authors have addressed most of the concerns I raised in the initial revision.

However, I still have some issues with Figure 1 and 2, and the way the authors define cell cycle stages. The EdU staining is very obvious and clear. In my view, the other cells are everything but S or M phase (M = condensed chromosomes). The one or two other GSCs in the images that aren’t encircled also fall into the non-S-phase and dot-like spectrosome category for the most part but in, for instance images Figure 1C and C’, have virtually undetectable CENP-C levels. I, therefore, do not see how the authors can quantify a subset of these cells in Figure 1H and conclude that the non-EdU cells that they have encircled are G2-prophase cells. Perhaps I am missing something but with the images and explanations provided this is not obvious to me.

To define G2 phase GSCs, we select cells that are EdU negative with a round spectrosome attached to the cap cell. Furthermore we use centromere positioning (on average 6 centromere foci spread throughout the nucleus) and chromosome morphology (uncondensed, based on DAPI staining) to define this stage. Therefore, we are confident that these cells are neither in S phase nor in mitosis (with the exception of very early prophase). In our previous publication in JCB (Dattoli et al. 2020), we failed to isolate G1 phase GSCs and concluded that this phase must be very short in this cell type. Indeed, we captured telophase nuclei with clustered centromeres that were already positive for EdU, meaning that these cells enter almost immediately from mitosis into S phase. In summary, we believe that these criteria are sufficient to identify G2/prophase GSCs.

With respect to Figure 1C and 1C’, the images presented are projections of the number of z stacks that specifically capture the spectrosome and all centromere foci of the circled GSC. The other GSCs in the images that are not circled are not in the same plane and therefore not all centromeres are visible. We have added this information to the legends of Figure 1, 2 and 3 (highlighted in blue text on p12 and p13): *The circled GSC is a projection of z-stacks that displays the spectrosome morphology (round or bridged) and all centromere foci of that specific cell.*

There is also one more concern with the HA-CENP-C overexpression. It does not affect CID assembly and does not rescue the germ line tumor phenotype. The results that overexpression shows a rescue of the differentiation defects and GSC loss (Fig 4) suggests that it is functional but it would be nice to see this with a CENP-C mutant. Perhaps this has been done by the source of the flies (McKim lab). There are many examples of tagged proteins that localize properly but are non-functional in rescue experiments. If this isn’t possible the authors should include a statement that they cannot fully rule out that the tagged protein is fully functional.

The reviewer makes a good point. Aside from our RNAi rescue experiments, we have not tested functionality of the HA-CENP-A line. We are not aware that the McKim lab has tested this either at this point (they have not yet published this fly line). Therefore, we have added the following statement to the results section on p8 (top, blue text): *We also cannot exclude the possibility that the rescue was incomplete as the HA-CENP-C protein is not fully functional.*
